# Longitudinal changes in Stress Responses, Stressors, and Social Supports among adolescent students: A latent growth curve modeling approach

**DOI:** 10.1002/pcn5.70244

**Published:** 2025-11-17

**Authors:** Miyuki Furukawa, Ayako Tsuchiya, Yurika Namihira, Yoshikazu Noda, Seiichiro Hori, Takako Koshiba, Hironori Shimada, Eiji Shimizu

**Affiliations:** ^1^ United Graduate School of Child Development, The University of Osaka, Kanazawa University, Hamamatsu University School of Medicine Chiba University and University of Fukui Osaka Japan; ^2^ Research Center for Child Mental Development Chiba University Chiba Japan; ^3^ Department of Nursing Josai International University Chiba Japan; ^4^ Department of Nursing, Faculty of Human Care at Makuhari Tohto University Chiba Japan; ^5^ Faculty of Human Sciences Waseda University Saitama Japan; ^6^ Department of Cognitive Behavioral Physiology, Graduate School of Medicine Chiba University Chiba Japan

**Keywords:** adolescent mental health, adolescent psychology, latent growth curve analysis, school stress assessment, stress inventory assessment

## Abstract

**Aim:**

To examine longitudinal changes in stress responses (SR), stressors (ST), and social supports (SS) among high school students using latent growth curve modeling.

**Background:**

This study investigated patterns of stress responses (SR), stressors (ST), and social supports (SS) among high school students over a three‐year period, using data from a web‐based stress check system.

**Methods:**

After ethical review approval, informed consent was obtained from both students and their parents. A longitudinal analysis was conducted using data collected from high school students (*n* = 605) over three consecutive years, from their first year (10th grade) to their third year (12th grade). High school students were assessed using the Public Health Research Foundation‐Type Stress Inventory via the web each year for three years. This inventory consists of items assessing SR, ST, and SS. Data were analyzed using latent growth curve modeling (LGCM).

**Results:**

In the LGCM, both the intercept (first‐year level) and slope of ST significantly predicted higher SR in the third year (*β* = 0.591 and 0.916, respectively; *p* < 0.001). By contrast, the intercept of SS (first‐year level) significantly predicted lower SR in the third year (*β* = –0.279, *p* < 0.001). Within the SS subscales, the intercept of support from friends significantly predicted reductions in all three SR subscales in the third year: depression/anxiety (*β* = –0.248, *p* < 0.05), irritability/anger (*β* = –0.254, *p* < 0.01), and helplessness (*β* = –0.223, *p* < 0.05).

**Conclusions:**

Our findings suggest the utility of a web‐based stress check administered in the first year of high school to assess SR, ST, and SS.

## INTRODUCTION

According to the World Health Organization (WHO), more than 13% of adolescents aged 10 to 19 are affected by some form of mental disorder, underscoring adolescent mental health as a pressing global public health concern.^1^ In Japan, this issue is also becoming increasingly serious. The Ministry of Education, Culture, Sports, Science and Technology (MEXT, 2024) reported a record high of 68,770 high school students experiencing school refusal in fiscal year 2023, accounting for 2.4% of the total student population. School non‐attendance is defined as prolonged non‐attendance due to psychological or social factors and has been reported to be associated with helplessness, irregular daily rhythm, and anxiety and depression.^2^

Adolescence is a period marked by rapid developmental changes and increasing demands for social adaptation, with experiences during this stage shown to have lasting effects on health across the lifespan.^3^ Adolescents are exposed to various psychosocial stressors on a daily basis. These stressors significantly impact both mental and physical health and may also impair school adaptation.^4^

Stress is defined as a process by which an individual responds physiologically and psychologically to demands (stimuli) arising from internal or external sources.^5^ The mental and physical changes that occur during this process are referred to as stress responses.^5^, ^6^ These responses may include symptoms, such as depression, anxiety, helplessness, anger, sleep disturbances, and somatic complaints. The stimuli that trigger these responses are known as stressors. In the school context, major stressors.^7^, ^8^, ^9^ include academic pressure,^10^ uncertainty regarding career paths,^11^ issues related to teachers,^9^ and instability at home.^12^ By contrast, social support—defined as emotional and instrumental assistance provided by others—plays a crucial role as a buffering factor that mitigates the adverse effects of stress.^13^

Since 2015, the Japanese government has mandated the use of a workplace stress check system for adults, assessing three core components: stress responses, stressors, and social supports.^14^ However, there is currently no equivalent system for adolescents in educational settings. In response, we launched a web‐based stress check system in 2021 to facilitate the primary prevention of mental health issues among high school students. This system is based on the Public Health Research Foundation‐Type Stress Inventory.^15^ We previously reported stress patterns in students experiencing high stress or financial difficulties using latent class analysis.^16^

The integration of these three components in the PSI enables a deeper understanding of each student's circumstances within educational settings. This approach draws on the stress‐buffering model of social support proposed by Cohen and Wills^13^ and the cognitive appraisal theory of stress developed by Lazarus and Folkman.^5^

This study aimed to clarify patterns of change over time in stress responses, stressors, and social support, as well as their interactions, using a latent growth curve model (LGCM) based on three years of longitudinal stress check data from high school students.

## METHODS

### Procedure

This study was approved by the Research Ethics Committee of the Chiba University Graduate School of Medicine (ID: M10631). In 2022, prior to the commencement of the study, informed consent was obtained from both parents and students. Parents were provided with a detailed explanation of the study and subsequently gave their consent. Thereafter, students read the study description, gave their informed consent, and completed the stress check survey using their personal digital devices. Upon completion, students were immediately able to review their stress status via the web platform.

### Participants

This study employed longitudinal survey data collected annually from September 2022 to December 2024, targeting high school students (aged 16–18 years) enrolled in public schools in a municipality near Tokyo in Japan. The first wave of data collection (Time 1) was conducted between September and December 2022, when participants were in their first year of high school (aged 15–16). The second wave (Time 2) was carried out between September and December 2023, during their second year (aged 16–17). The third wave (Time 3) took place between September and December 2024, during their third year (aged 17–18).

This study was originally conducted as an annual cross‐sectional study, and participants voluntarily agreed to participate each time. Therefore, there was no system to ensure that all students would continue to participate across the three years. The number of participants was limited to this number because the data from the three consecutive years of the cross‐sectional studies were analyzed as three‐year longitudinal data.

**Figure 1:**
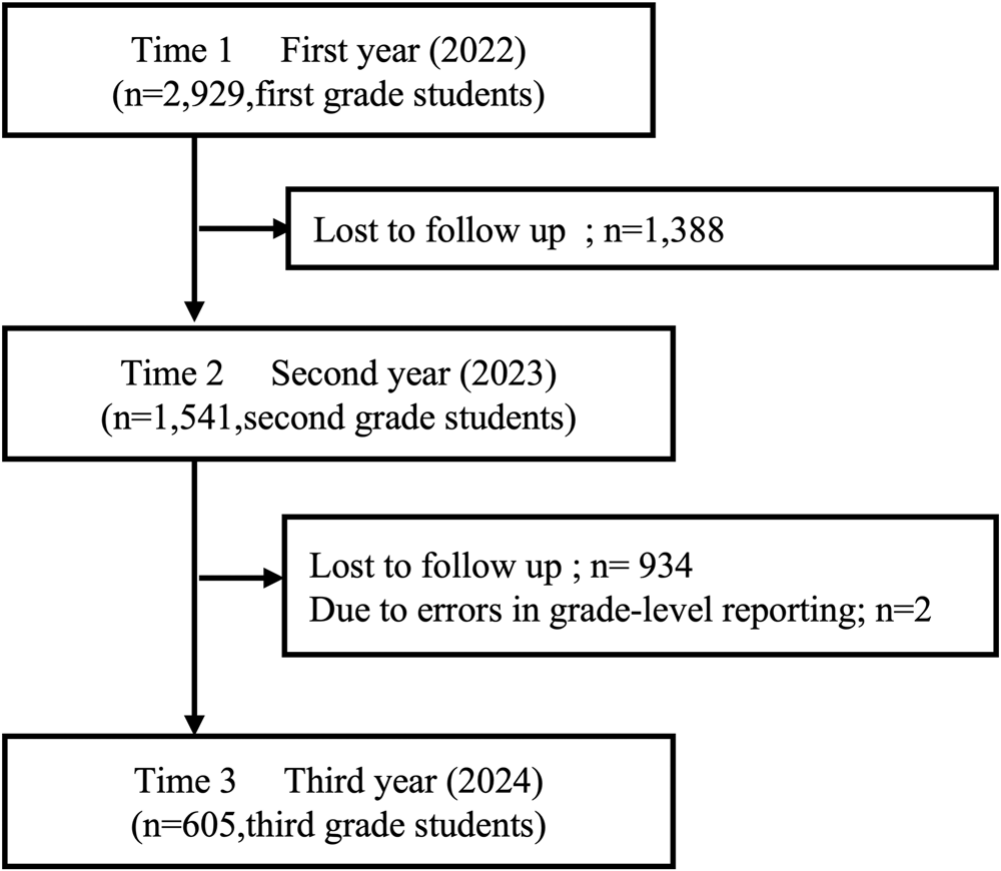
Flow diagram of participants.

In Time 1 (First year, 2022), of the 7064 students in all grades, including first, second, and third graders, data were available for 2929 first‐grade students. In Time 2 (Second year, 2023), of the 31,181 students in all grades, including first, second, and third graders, data were available for 10,306 second‐grade students. Of these, 1541 second‐grade students participated in both Time 1 and Time 2. In Time 3 (Third year, 2024), of the 41,718 students in all grades, including first, second, and third graders, data were available for 12,587 third‐grade students. Of these, 607 third‐grade students participated continuously across all three years (Time 1, Time 2, and Time 3). After excluding two students due to errors in grade‐level reporting, the final analytic sample consisted of 605 participants (Figure 1).

### Measures

#### The Public Health Research Foundation‐Type Stress Inventory (PSI)

The Public Health Research Foundation‐Type Stress Inventory (PSI) is a comprehensive tool developed by Sakano and colleagues to assess the daily mental health of children^17^. Further information regarding the PSI is provided in our previous study (under review, the manuscript revised for publication in Psychiatry and Clinical Neurosciences Reports, by Namihira et al.)^16^. The version specifically developed for high school students was employed in this study. Previous validation studies have demonstrated satisfactory internal consistency (Cronbach's *α* > 0.80) and construct validity, with significant correlations with related psychological measures, such as emotional intelligence and mental health indicators^18^, ^19^. The PSI comprises three main components: 15 items on stress responses (SR), 16 items on stressors (ST), and 12 items on social supports (SS). It is a four‐point scale with scores ranging from 0 to 3.

The SR component includes three subscales: depression and anxiety (DEP; items 1, 4, 7, 10, 13), irritability (IRR; items 2, 5, 8, 11, 14), and helplessness (HEL; items 3, 6, 9, 12, 15). The ST component comprises four subscales: academic stressors (ACA; items 3, 7, 11, 15), career stressors (CAR; items 4, 8, 12, 16), stressors related to teachers (TEA; items 1, 5, 9, 13), and stressors related to friends (FRI; items 2, 6, 10, 14). The SS component includes three subscales: support at home (SHOM; items 1, 4, 7, 10), support from school (SSCH; items 2, 5, 8, 11), and support from friends (SFRI; items 3, 6, 9, 12). The reliability of each scale was assessed using Cronbach's alpha (α)^20^.

### Statistical analysis

Descriptive statistics were first calculated for each variable. Means and standard deviations were computed for the SR, ST, and SS components and their subscales. Repeated measures analysis of variance (ANOVA) was then conducted to examine significant changes in SR, ST, and SS across the three time points. Post hoc tests were performed to assess the significance of differences between time points. Subscale comparisons at each time point were conducted to examine within‐group differences. Additionally, Pearson's product‐moment correlation coefficients were calculated at each time point to examine the associations among SR, ST, and SS.

In addition, gender differences in SR, ST, and SS were examined in the same procedures.

To analyse longitudinal changes in the variables over the three years, latent growth curve modeling (LGCM) was employed. In this framework, intercepts and slopes were specified as latent variables, enabling the simultaneous estimation of both intra‐individual change and inter‐individual variability^21^.

Model fit was evaluated using the following indices: chi‐square to degrees of freedom ratio (χ²/df), comparative fit index (CFI), Tucker–Lewis Index (TLI), root mean square error of approximation (RMSEA), and standardized root mean square residual (SRMR). Based on Hu and Bentler (1999), model fit was considered acceptable if CFI and TLI values were ≥0.90 and good if ≥0.95; RMSEA values < 0.08 and SRMR values <0.06 also indicated good fit^22^.

We first constructed unconditional linear growth models for SR, ST, and SS to examine general developmental trends. Subsequently, separate models were estimated for each subscale to explore specific trajectories. A parallel LGCM was also conducted, incorporating ST and SS as covariates to estimate their longitudinal associations with SR). Gender was included as a time‐invariant covariate to examine the extent to which the intercepts and slopes of ST and SS predicted the developmental trajectories of SR over time.

All LGCM analyses were performed using Mplus version 8.4 (Muthén & Muthén, Los Angeles, CA, USA)^23^. ANOVA and correlation analyses were conducted using Stata version 18 (Stata Corp LP, College Station, TX, USA)^24^. A two‐tailed significance level of *p* < 0.05 was adopted for all statistical tests.

## RESULTS

Data from 605 students who participated in all three waves of the survey over the three‐year period were analyzed. Of these, 267 (44.1%) identified as male, 305 (50.4%) as female, and 33 (5.5%) selected “other.” The “other” category included students who chose “other” or left the gender field blank. Gender was included as a covariate in all latent growth curve models to account for potential sex differences.

Cronbach's alpha (α) values indicated high internal consistency across all scales: SR, *α* = 0.94–0.95; ST, *α* = 0.79–0.83; and SS, *α* = 0.93–0.95.

### Longitudinal changes and subscale comparisons

The means and standard deviations for the observed variables at each time point are presented in Table 1. To examine longitudinal changes from Time 1 (T1) to Time 3 (T3) in the subscales of SR, ST, and SS, repeated measures analysis of variance (ANOVA) was conducted, followed by post hoc comparisons. The results revealed significant differences across time points for all three constructs (*ps* < 0.001; Table 1).

**Table 1:** Comparison of mean and standard deviation of variable at three time points (*n* ＝ 605).

		T1 (First grade)	T2 (Second grade)	T3 (Third grade)	Longitudinal changes			
		M (SD)	M (SD)	M (SD)	*p* value	Post hoc comparisons		
						t1vst2	t2vst3	t1vst3
SR		15.67 (10.59)	16.99 (11.59)	16.67 (11.84)	[***]	[***]	n.s.	[*]
ST		10.83 (6.19)	11.95 (6.49)	11.61 (6.93)	[***]	[***]	n.s.	[***]
SS		23.78 (8.51)	23.89 (8.32)	25.10 (9.00)	[***]	n.s.	[***]	[***]
SR								
	DEP	5.04 (3.92)	5.28 (4.20)	5.38 (4.23)	[***]	n.s.	n.s.	n.s.
	IRR	4.44 (3.88)	4.88 (4.18)	4.72 (4.28)	[***]	[***]	n.s.	n.s.
	HEL	6.18 (3.91)	6.83 (4.21)	6.57 (4.36)	[***]	[***]	n.s.	[**]
ST								
	ACA	5.09 (2.94)	5.00 (3.06)	4.11 (3.12)	[***]	n.s.	[***]	[***]
	CAR	3.73 (2.44)	4.95 (2.51)	5.62 (2.89)	[***]	[***]	[***]	[***]
	TEA	1.19 (1.94)	1.13 (2.00)	0.98 (1.83)	[***]	n.s.	n.s.	[**]
	FRI	0.83 (1.49)	0.88 (1.60)	0.90 (1.70)	[***]	n.s.	n.s.	n.s.
SS								
	SSCH	6.83 (3.41)	6.98 (3.28)	7.67 (3.45)	[***]	n.s.	[***]	[***]
	SFRI	8.69 (3.05)	8.57 (3.08)	8.85 (3.18)	[***]	n.s.	[**]	[*]
	SHOM	8.26 (3.48)	8.35 (3.41)	8.58 (3.50)	[***]	n.s.	[*]	[*]

Abbreviations: ACA, academic stressors; CAR, career stressors; DEP, depression and anxiety; FRI, stressors related to friends; HEL, helplessness; IRR, irritability; M, mean; SD, standard deviation; SFRI, support from friends; SHOM, support at home; SR, stress responses; SS, social supports; SSCH, support from school; ST, stressors; TEA, stressors related to teachers.

* *p* < 0.05

** *p* < 0.01

*** *p* < 0.001, n.s.: not significant.

Specifically, mean scores for both SR and ST increased significantly from T1 to T2 (*p* < .001), whereas SS increased significantly from T2 to T3 (*p* < 0.001). Within the SR subscales, irritability (IRR) and helplessness (HEL) showed significant increases from T1 to T2 (*p* < 0.001). Regarding the ST subscales, academic stressors (ACA) decreased significantly from T2 to T3 (*p* < 0.001), while career stressors (CAR) increased significantly from T1 to T2 and again from T2 to T3 (*ps* < 0.001).

All three SS subscales—support from school (SSCH), support from friends (SFRI), and support at home (SHOM)—increased significantly from T2 to T3 (SSCH; *p* < 0.001, SFRI; *p* < 0.01, SHOM; *p* < 0.05) (Table 1).

Subscale comparisons at each time point were also conducted. For the SR subscales—depression/anxiety (DEP), irritability (IRR), and helplessness (HEL)—significant differences were found at all time points. For example, at T3, a significant overall difference was observed (F (30, 574) = 55.17, *p* < 0.001), and Bonferroni‐corrected post hoc comparisons indicated that HEL scores were significantly higher than those for DEP and IRR (*ps* < 0.001) (data not shown).

Similarly, for the ST subscales—ACA, CAR, stressors related to teachers (TEA), and stressors related to friends (FRI)—significant differences were observed at each time point (e.g., T3: F (32, 575) = 8.36, *p* < 0.001). Bonferroni‐corrected post hoc tests revealed that ACA and CAR were perceived as significantly more stressful than TEA and FRI (*ps* < 0.001). For the SS subscales—SFRI, SSCH, and SHOM—significant differences were also observed at all time points (e.g., T3: F (24, 580) = 29.74, *p* < 0.001). Post hoc comparisons indicated that support from friends (SFRI) was rated significantly higher than both support from school (SSCH) and support at home (SHOM) (*ps* < 0.001).

Finally, gender comparisons at each time point were also conducted. For SR, significant gender differences were found at all time points (T1, T2, and T3) (*ps* < 0.001). Post‐hoc comparisons revealed that females scored significantly higher than males at all time points (T1, T2, and T3) (*ps* < 0.001), and the ‘other’ group scored significantly higher than males at T1 (*p* < 0.01). For ST, significant gender differences were found at T1 (*p* < 0.01) and T2 (*p* < 0.001). Post‐hoc comparisons showed that females scored significantly higher than males at T1 (*p* < 0.01) and T2 (*p* < 0.001). For SS, no significant gender differences were found at any time points (T1, T2, or T3) (Table 2).

**Table 2:** Comparison of gender differences in mean and standard deviation of SR, ST, and SS at three time points (*n* = 605).

Variable	Gender	T1 (First grade)	T2 (Second grade)	T3 (Third grade)			T1	T2	T3
		M (SD)	M (SD)	M (SD)		Gender differences			
SR	male	12.97 (9.61)	14.35 (11.15)	14.14 (11.44)	*p* value		[***]	[***]	[***]
	female	17.55 (10.66)	19.06 (11.53)	18.63 (11.75)	post‐hoc comparisons	male vs female	[***]	[***]	[***]
	other	20.06 (12.39)	19.21 (11.62)	19.50 (12.39)		female vs other	n.s.	n.s.	n.s.
						male vs other	[**]	n.s.	n.s.
ST	male	9.89 (9.61)	10.70 (11.15)	10.95 (11.44)	*p* value		[**]	[***]	n.s.
	female	11.54 (10.66)	13.00 (11.53)	12.10 (11.75)	post‐hoc comparisons	male vs female	[**]	[***]	n.s.
	other	11.88 (12.39)	12.42 (11.62)	12.68 (12.39)		female vs other	n.s.	n.s.	n.s.
						male vs other	n.s.	n.s.	n.s.
SS	male	23.82 (9.61)	24.22 (11.15)	25.28 (11.44)	*p* value		n.s.	n.s.	n.s.
	female	24.05 (10.66)	23.94 (11.53)	25.20 (11.75)	post‐hoc comparisons	male vs female	n.s.	n.s.	n.s.
	other	21.00 (12.39)	20.73 (11.62)	22.25 (12.39)		female vs other	n.s.	n.s.	n.s.
						male vs other	n.s.	n.s.	n.s.

Abbreviations: M, mean; SD, standard deviation; SR, stress responses; SS, social supports; ST, stressors.

** *p* < 0.01

*** *p* < 0.001, n.s.: not significant.

### Associations among SR, ST, and SS

To examine the associations among SR, ST, and SS, Pearson's correlation coefficients were calculated at each time point (Table S1). Results showed a significant positive correlation between SR and ST (e.g., T3: *r* = 0.62, *p* < 0.001), and a moderate negative correlation between SR and SS (e.g., T3: *r* = −0.44, *p* < 0.001).

Each SS subscale—SSCH, SFRI, and SHOM—showed moderate negative correlations with all SR subscales (DEP, IRR, and HEL) at each time point (all *ps* < 0.001).

### Latent growth curve modeling of longitudinal changes in SR, ST, and SS

To examine how changes in ST and SS influenced SR, a Latent Growth Curve Model (LGCM) was applied to the three time points of data. LGCM allows for the estimation of individual‐level growth trajectories by estimating intercepts and slopes as latent variables.^25^ (Muthén & Curran, 1997).

**Figure 2:**
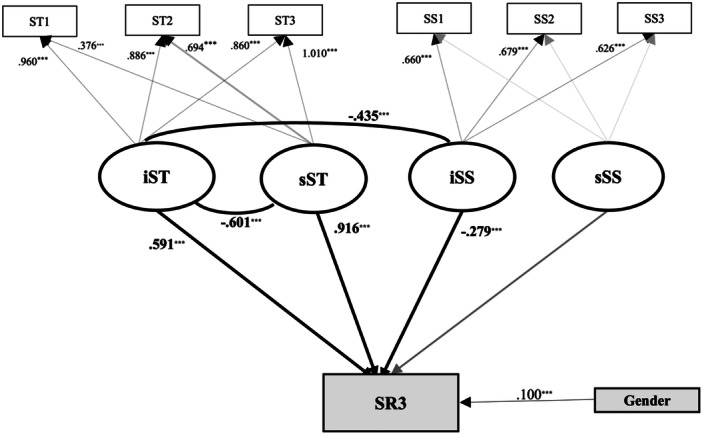
Latent growth curve model predicting stress responses (SR) at the 3rd year from changes in stressors (ST) and social supports (SS). Model fit indices: CFI = 0.955, TLI = 0.930, RMSEA = 0.059, SRMR = 0.048. ST1 (stressors in the 1st year), ST2 (stressors in the 2nd year), ST3 (stressors in the 3rd year), SS1 (social supports in the 1st year), SS2 (social supports in the 2nd year), ST3 (social supports in the 3rd year), iST (the intercept of ST), sST (the slope of ST), iSS (the intercept of SS), sSS (the slope of SS), and SR3 (stress responses in the 3rd year).***p < 0.001.

A parallel LGCM was constructed (Figure 2), with SR at T3 as the outcome and ST and SS as covariates. The linear growth model demonstrated a good fit to the data (CFI = 0.955, TLI = 0.930, RMSEA = 0.059, SRMR = 0.048).

SR at T3 was significantly predicted by both the intercept (*β* = 0.591, *p* < 0.001) and slope (*β* = 0.916, *p* < 0.001) of ST, suggesting that both the initial level of stressors (at T1) and their rate of increase over time contributed to elevated SR by the third year.

By contrast, the intercept of SS significantly predicted a decrease in SR at T3 (*β* = −0.279, *p* < 0.001), indicating that higher levels of social supports in the first year were associated with lower stress responses in the third year.

A significant negative covariance was also observed between the intercepts of ST and SS (*r* = −0.435, *p* < 0.001), suggesting that participants who reported higher social support at T1 also tended to perceive lower levels of stressors at that time.

### Latent growth curve modeling of longitudinal changes in Subscales of SR, ST, and SS

Next, we examined longitudinal changes across the three time points for subscales of SR, ST, SS.

Separate latent growth curve models (LGCMs) were constructed for each SR subscale—DEP, IRR, and HEL—using T3 scores as outcome variables and ST as covariates. An initial parallel LGCM, which included multiple ST subscales as covariates simultaneously, demonstrated poor fit under a linear growth model. Consequently, separate models were constructed by entering each ST subscale—academic stressors (ACA), career stressors (CAR), stressors related to teachers (TEA), and stressors related to friends (FRI)—individually as covariates. All models, except for the one including career stressors (CAR), demonstrated acceptable fit (Table S2). The results of these LGCMs are presented in Table 3.

**Table 3:** Results of parallel LGCM with covariates.

		Mean		DEP3 on		IRR3 on		HEL3 on	
Model	Covariates	Intercept	Slope	Intercept	Slope	Intercept	Slope	Intercept	Slope
**M1**	**ACA**	2.068***	−0.519***	0.485	0.645***	0.449***	0.659	0.592***	0.768***
**M2**	**TEA**	0.750***	−0.170*	0.548***	0.601***	0.671**	0.732*	0.489***	0.553***
**M3**	**FRI**	0.652***	0.082	0.442**	0.521**	0.441*	0.661***	0.407***	0.469***

Abbreviations: ACA, academic stressors; DEP, depression and anxiety; FRI, stressors related to friends; HEL, helplessness; IRR, irritability; M, mean; TEA, stressors related to teachers.

* *p* < 0.05

** *p* < 0.01

*** *p* < 0.001.

In Model 1 (M1), both the intercept and slope of ACA significantly and positively predicted all three SR subscales—DEP, IRR, and HEL—at T3 (*ps* < 0.05).

In Model 2 (M2), the intercept and slope of TEA were also significant positive predictors of all SR subscales at T3 (*ps* < 0.05).

In Model 3 (M3), both the intercept and slope of stressors related to friends (FRI) significantly and positively predicted T3 levels of DEP, IRR, and HEL (*ps* < 0.05).

Next, LGCMs were constructed to examine the effects of both ST and SS on SR subscales at T3. A model incorporating all three SS subscales simultaneously as covariates failed to meet the criteria for adequate model fit. Thus, separate models were developed by entering each SS subscale—support from school (SSCH), support from friends (SFRI), and support at home (SHOM)—individually, as a covariate. All these models demonstrated good fit (Table S3), and the LGCM results are presented in Table 4.

**Table 4:** Parallel LGCM with covariates of stressor and social supports.

		DEP3 on		IRR3 on		HEL3 on	
Model	Variables	Intercept	Slope	Intercept	Slope	Intercept	Slope
M4	ACA	0.275	0.370	0.239	0.377	0.427*	0.549*
	SSCH	−0.437*	−0.553	−0.442*	−0.549	−0.368**	−0.424
**M5**	**ACA**	**0.360** **	**0.529** ***	**0.348** **	**0.56** ***	**0.466** ***	**0.644** *
	**SFRI**	**−0.248** *	**−0.346** **	**−0.254** **	**−0.283** *	**−0.223** *	**−0.344** *
M6	ACA	0.448***	0.746*	0.393***	0.665**	0.566***	0.852*
	SHOM	−0.249***	—	−0.256***	—	−0.189***	—
M7	CAR	0.418	0.489	0.29	0.351	0.525	0.591
	SSCH	−0.559***	−0.651	−0.56***	−0.682	−0.544***	−0.579
**M8**	**CAR**	**0.498** ***	**0.617** ***	**0.393** ***	**0.512** ***	**0.572** ***	**0.672** *
	**SFRI**	**−0.341** *	**−0.416** **	**−0.342** *	**−0.367** **	**−0.331** *	**−0.449** *
M9	CAR	0.539***	0.893	0.419**	0.773	0.627***	0.985
	SHOM	−0.369***	—	−0.366***	—	−0.335***	—
M10	FRI	0.364*	0.012	0.369**	0.293	0.35	0.038
	SSCH	−0.456	−0.833	−0.409	−0.609	−0.454	−0.828
M11	FRI	0.463	0.226	0.356	0.588	0.511	0.069
	SFRI	−0.112	−0.491	−0.217	−0.650	−0.057	−0.650
M12	FRI	0.156	0.608	0.255	0.660**	0.129	0.604
	SHOM	−0.323**	—	−0.271**	—	−0.305*	—

Abbreviations: ACA, academic stressors; CAR, career stressors; FRI, stressors related to friends; SHOM, support at home, SSCH, support from school, SFRI, support from friends; TEA, stressors related to teachers.

* *p* < 0.05

** *p* < 0.01

*** *p* < 0.001.

**Figure 3:**
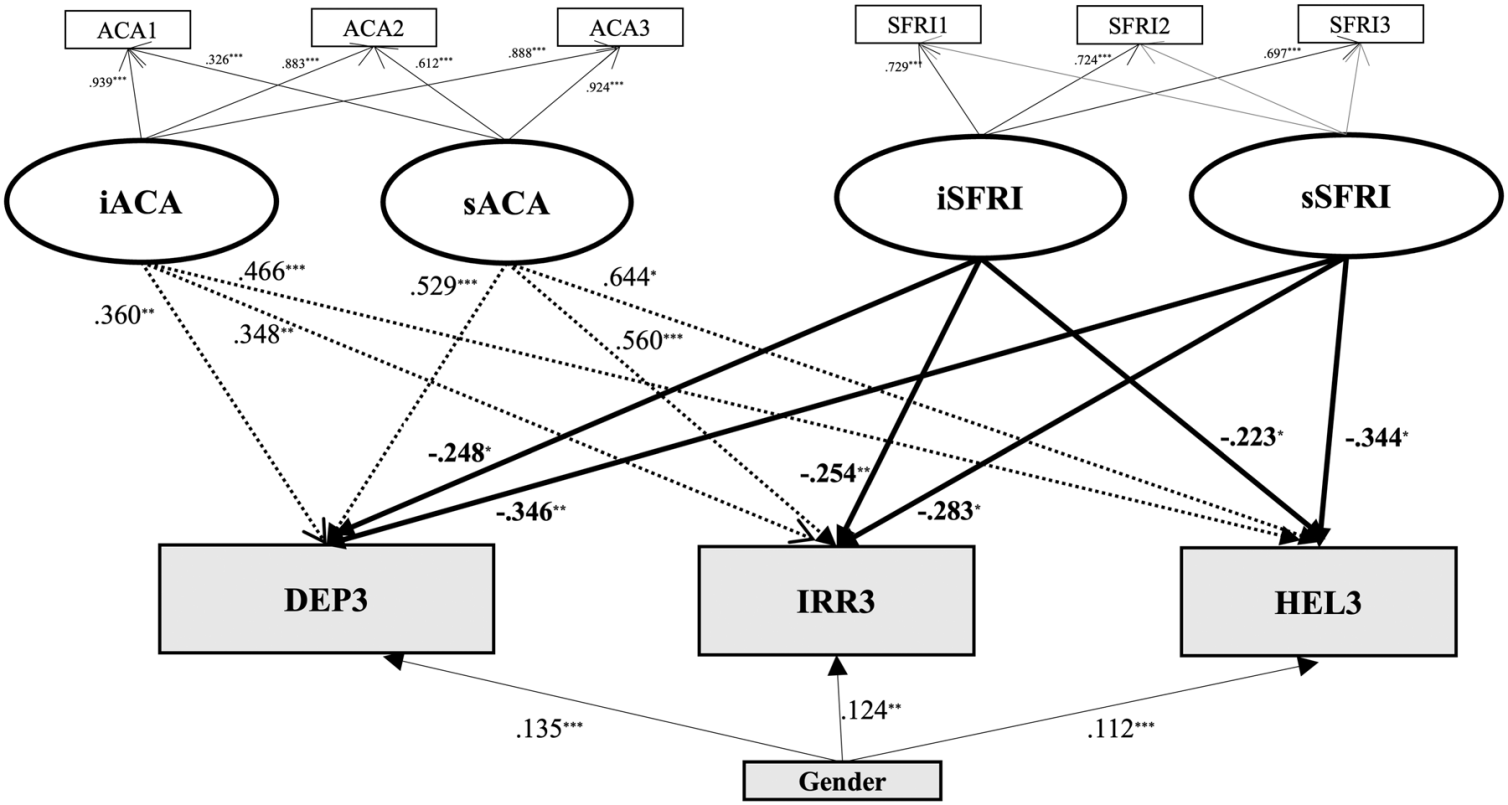
Latent growth curve model (Model 5) predicting depression/anxiety (DEP), irritability (IRR), and helplessness (HEL) in the 3rd year from changes in academic stressors (ACA), support from friends (SFRI), and gender. Model fit indices: CFI = 0.986, TLI = 0.967, RMSEA = 0.049, SRMR = 0.037. ACA1 (academic stressors in the 1st year), ACA2 (academic stressors in the 2nd year), ACA3 (academic stressors in the 3rd year), SFRI1 (support from friends in the 1st year), SFRI 2 (support from friends in the 2nd year), SFRI 3 (support from friends in the 3rd year), iACA (the intercept of academic stressors), sACA (the slope of academic stressors), iSFRI (the intercept of support from friends), sSFRI (the slope of support from friends), DEP3 (depression/anxiety in the 3rd year), IRR3 (irritability in the 3rd year), and HEL3 (helplessness in the 3rd year). *p < 0.05, **p < 0.01, ***p < 0.001.

Among Models 4 to 12, Model 5 (M5)—which included ACA and SFRI as covariates—yielded particularly noteworthy results (Figure 3). Both the intercept and slope of SFRI significantly and negatively predicted all three SR subscales at T3.

**Figure 4:**
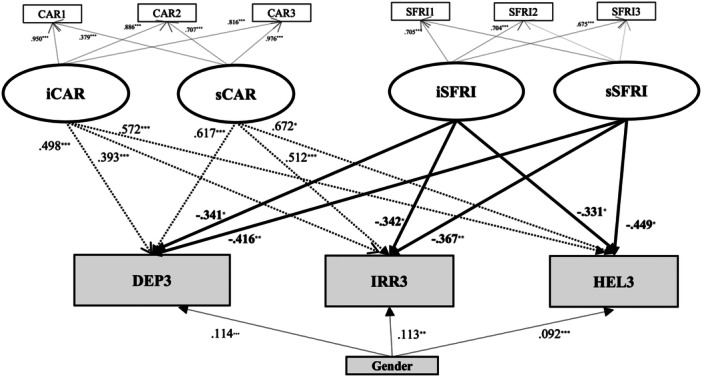
Latent growth curve model (Model 8) predicting depression/anxiety (DEP), irritability (IRR), and helplessness (HEL) at the 3rd year from changes in career stressors (CAR), support from friends (SFRI), and gender. Model fit indices: CFI = 0.976, TLI = 0.944, RMSEA = 0.061, SRMR = 0.049. CAR1 (career stressors in the 1st year), CAR2 (career stressors in the 2nd year), CAR3 (career stressors in the 3rd year), SFRI1 (support from friends in the 1st year), SFRI 2 (support from friends in the 2nd year), SFRI 3 (support from friends in the 3rd year), iCAR (the intercept of career stressors), sCAR (the slope of career stressors), iSFRI (the intercept of support from friends), sSFRI (the slope of support from friends), DEP3 (depression/anxiety in the 3rd year), IRR3 (irritability in the 3rd year), and HEL3 (helplessness in the 3rd year). *p < 0.05, **p < 0.01, ***p < 0.001.

Additionally, in models including gender as a covariate (coded as 1 = *male*, 2 = *female*, 3 = *other*), gender showed significant positive associations with all SR subscales at T3. Similarly, in Model 8 (M8), which included career stressors (CAR) and SFRI as covariates, both the intercept and slope of SFRI again significantly and negatively predicted DEP, IRR, and HEL at T3 (*ps* < 0.05; Figure 4).

## DISCUSSION

### Summary of Findings

This longitudinal study tracked changes in SR, ST, and SS over a three‐year period among 605 Japanese high school students. SR and ST significantly increased from T1 to T2, while SS significantly increased from T2 to T3. Among the SR subscales, HEL was perceived most strongly, followed by DEP and IRR. The most prominent stressors were ACA and CAR. By contrast, among SS subscales, SFRI was perceived most strongly.

The parallel LGCM revealed the following three main findings:

1. High levels of ST at T1 significantly predicted increased SR at T3, whereas higher levels of SS at T1 significantly suppressed SR at T3.
2. Among the types of ST, both academic and career‐related stressors at T1 significantly increased all three SR subscales at T3.
3. Regarding SS, the initial level and rate of increase in SFRI significantly reduced DEP, IRR, and HEL at T3.

### Interrelationships among Stress Reactions, Stressors, and Social Supports

These results emphasize the importance of providing social support early in the school experience.

They align with a four‐year longitudinal study by Rueger et al. (2021) conducted in the United States, which found that interpersonal stressors (school, family, and friends) predicted depressive and anxiety symptoms, while support from teachers, family, and friends mitigated those symptoms.^26^

### Changes in SR during High School

This study categorized SR into three subscales, with HEL consistently emerging as the most prominent. Prior research by Salmela‐Aro and Tynkkynen identified HEL as a core component of academic burnout.^27^ Academic stressors may reduce self‐efficacy, which in turn may heighten feelings of helplessness.

### Stressors in School Life and SR

Academic and career‐related stressors were rated significantly higher than other types. While academic stressors decreased from T2 to T3, career‐related stressors increased significantly over a three‐year period. These trends are consistent with findings from a Finnish longitudinal study, which reported that academic pressure tends to decrease over time due to increased autonomy and clearer goals, whereas uncertainty regarding career pathways tends to intensify.^28^

Parallel LGCMs further demonstrated that both the intercept and slope of academic stressors significantly predicted all three SR subscales at T3. These findings corroborate earlier research by Salmela‐Aro and Tynkkynen and Jung et al., which indicated that academic stressors contribute to helplessness via diminished self‐efficacy.^27^, ^29^

### The buffering role of social support

Support from friends significantly moderated the impact of both academic and career stressors on all three SR subscales. These results emphasize the protective role of peer support. Fitzpatrick et al. reported similar findings in a U.S. cohort of 1348 adolescents, where higher levels of support from friends were associated with fewer depressive and anxiety symptoms.^30^

This study extends these findings to include helplessness and irritability IRR. Gentzler et al.^31^ also highlighted the influence of friendship quality on anger regulation. In China, Shao et al.^32^ and Gao et al.^33^ found that peer relationships enhanced both academic motivation and performance. Although these earlier studies were cross‐sectional, our longitudinal findings suggest similar mechanisms. The results indicate that early interventions promoting peer support, particularly in the first year of high school, can effectively reduce SR. This supports the value of primary preventive interventions for mental health. Notably, Witvliet et al. found that support from friends was protective against internalizing symptoms (e.g., depression, anxiety), but less so for externalizing problems (e.g., aggression).^34^

However, our study showed that support from friends significantly reduced irritability, suggesting a broader protective role during late adolescence. Lastly, our findings indicate that early difficulties with stressors related to friends and increased academic stressors may contribute to rising levels of depression/anxiety. This is consistent with Shore et al., who identified peer difficulties as a contributing factor to adolescent depression.^35^ Friendship is widely recognized as important during adolescence. This is because friendship has two sides: it can function both as stressors related to friends and as social support from friends.

### Gender differences in SR

LGCMs incorporating gender as a covariate revealed significant effects across all SR subscales at T3. Female and non‐binary/undisclosed students exhibited higher SR scores than male students, suggesting that psychological vulnerability may differ by gender.

These results are consistent with a systematic review by Shore et al., which identified gender (particularly female), social context, and heightened stress reactivity as predictors of adolescent depression.^35^ Similar patterns were observed in a Tunisian cohort, where female students exhibited higher levels of depression and anxiety, particularly when experiencing psychotic symptoms.^36^

Importantly, this study highlights not only increased vulnerability among female students but also elevated SR among gender‐diverse youth—a group often underrepresented in existing research. These findings underscore the need for more inclusive mental health support frameworks that consider gender diversity.

### Implications for practice

For teachers and other school staff (e.g., teachers, counselors, and school nurses), these findings offer practical implications. Monitoring students' stress trajectories—especially symptoms such as helplessness and depression/anxiety—can help identify critical points for early intervention. In particular, understanding the strong influence of academic and career stressors, as well as the buffering effect of peer support, may inform improvements to career and learning guidance and the management of friend‐related stressors.

### Limitations and future directions

Several limitations should be acknowledged. First, the sample size was modest. Larger‐scale cohort studies involving all students are necessary for broader generalization.^37^ Second, the study did not stratify participants by region, academic level, school type (e.g., general vs. vocational), or socioeconomic status, thereby limiting the granularity of analysis. Future research should employ random sampling for broader applicability.^38^

Third, this study did not assess coping strategies or the quality of relationships with teachers and family members, which are potential mediators or moderators. Future studies should incorporate these variables for more comprehensive modeling.^4^, ^39^

Fourth, as noted in the Introduction, school non‐attendance is a serious issue during adolescence in Japan. This study targeted all enrolled students, and because the survey was completed using students' personal digital devices, it was possible for students to complete it even if they were absent on the day of implementation. Students with school non‐attendance were therefore not necessarily excluded. Indeed, some participating schools reported that students experiencing school non‐attendance had completed the survey.

However, at the time of analysis, we lacked data from the schools regarding the number or percentage of respondents who were experiencing school non‐attendance. In addition, we did not extract and analyze data specifically from students with school non‐attendance; therefore, we were unable to fully examine the potential link between helplessness and school non‐attendance. Consequently, we did not extract or analyze data specifically for this subgroup, preventing a full examination of the potential link between study variables, including helplessness and school non‐attendance. This represents a limitation of the current study and an important avenue for future research.

## CONCLUSION

This study followed changes in SR, ST, and SS over three years and investigated their interrelations using parallel latent growth curve modeling. The results showed that increased academic and career stressors in the first year significantly predicted higher SR in the third year, while support from friends (SFRI) in the first year was associated with lower SR. These findings highlight the importance of reducing ST and enhancing SS early in high school.

## AUTHOR CONTRIBUTIONS

Miyuki Furukawa, Yurika Namihira, Ayako Tsuchiya, and Eiji Shimizu collected the data. Miyuki Furukawa, Yoshikazu Noda, and Yurika Namihira performed the statistical analyses and interpreted the data. Yoshikazu Noda, Hironori Shimada, and Eiji Shimizu reviewed and edited the manuscript. Takako Koshiba, Seiichiro Hori and Eiji Shimizu supervised the project. All authors contributed to and approved the final version of the manuscript.

## CONFLICT OF INTEREST STATEMENT

The authors declare no conflicts of interest.

## ETHICS APPROVAL STATEMENT

This study was approved by the Ethics Committee of Chiba University (ID: M10631). All participants and their guardians provided informed consent.

## PATIENT CONSENT STATEMENT

Informed consents were obtained from the guardians before participation. Students were also informed about the study and provided their own consents before completing the survey on their digital devices.

## CLINICAL TRIAL REGISTRATION

N/A.

## Supporting information

Supplementery Table.

## Data Availability

The data that support the findings of this study are available on request from the corresponding author. The data are not publicly available due to privacy or ethical restrictions.
